# Indoxyl Sulfate, a Tubular Toxin, Contributes to the Development of Chronic Kidney Disease

**DOI:** 10.3390/toxins12110684

**Published:** 2020-10-29

**Authors:** Tong-Hong Cheng, Ming-Chieh Ma, Min-Tser Liao, Cai-Mei Zheng, Kuo-Cheng Lu, Chun-Hou Liao, Yi-Chou Hou, Wen-Chih Liu, Chien-Lin Lu

**Affiliations:** 1 School of Medicine, Fu Jen Catholic University, New Taipei 242, Taiwan; Tonghong1108@yahoo.com.tw (T.-H.C.); med0041@mail.fju.edu.tw (M.-C.M.); liaoch22@gmail.com (C.-H.L.); athletics910@gmail.com (Y.-C.H.); 2 Department of Internal Medicine, Taoyuan Armed Forces General Hospital, Taoyuan 325, Taiwan; 3 Department of Pediatrics, Taoyuan Armed Forces General Hospital, Taoyuan 325, Taiwan; liaoped804h@yahoo.com.tw; 4 Department of Pediatrics, Tri-Service General Hospital, National Defense Medical Center, Taipei 114, Taiwan; 5 Division of Nephrology, Department of Internal Medicine, School of Medicine, College of Medicine, Taipei Medical University, Taipei 110, Taiwan; 11044@s.tmu.edu.tw; 6 Division of Nephrology, Department of Internal Medicine, Taipei Medical University Shuang Ho Hospital, New Taipei 235, Taiwan; 7 Taipei Medical University-Research Center of Urology and Kidney, Taipei Medical University, Taipei 110, Taiwan; 8 Division of Nephrology, Department of Medicine, Taipei Tzu Chi Hospital, Buddhist Tzu Chi Medical Foundation, New Taipei 231, Taiwan; kuochenglu@gmail.com; 9 Divisions of Urology, Department of Surgery, Cardinal Tien Hospital, New Taipei 23148, Taiwan; 10 Division of Nephrology, Department of Medicine, Cardinal-Tien Hospital, School of Medicine, Fu-Jen Catholic University, New Taipei 234, Taiwan; 11 Division of Nephrology, Department of Medicine, Taipei Hospital, Ministry of Health and Welfare, New Taipei 242, Taiwan; wayneliu55@gmail.com; 12 Division of Nephrology, Department of Medicine, Fu Jen Catholic University Hospital, New Taipei 242, Taiwan

**Keywords:** chronic kidney disease, indoxyl sulfate, oxidative stress, renal fibrosis, tubular injury

## Abstract

Indoxyl sulfate (IS), a uremic toxin, causes chronic kidney disease (CKD) progression via its tubulotoxicity. After cellular uptake, IS directly induces apoptotic and necrotic cell death of tubular cells. Additionally, IS increases oxidative stress and decreases antioxidant capacity, which are associated with tubulointerstitial injury. Injured tubular cells are a major source of transforming growth factor-β1 (TGF-β1), which induces myofibroblast transition from residual renal cells in damaged kidney, recruits inflammatory cells and thereby promotes extracellular matrix deposition in renal fibrosis. Moreover, IS upregulates signal transducers and activators of transcription 3 phosphorylation, followed by increases in TGF-β1, monocyte chemotactic protein-1 and α-smooth muscle actin production, which participate in interstitial inflammation, renal fibrosis and, consequently, CKD progression. Clinically, higher serum IS levels are independently associated with renal function decline and predict all-cause mortality in CKD. The poor removal of serum IS in conventional hemodialysis is also significantly associated with all-cause mortality and heart failure incidence in end-stage renal disease patients. Scavenging the IS precursor by AST-120 can markedly reduce tubular IS staining that attenuates renal tubular injury, ameliorates IS-induced oxidative stress and rescues antioxidant glutathione activity in tubular epithelial cells, thereby providing a protective role against tubular injury and ultimately retarding renal function decline.

## 1. Overview of Uremic Toxin Indoxyl Sulfate

A substantial number of compounds that are normally excreted by healthy kidneys are accumulated during renal function deterioration, in which case they are called uremic toxins. A uremic toxin can be categorized by its molecular weight (MW), including free water-soluble low-molecular-weight solutes (MW < 500 D), small protein-bound solutes (mostly MW < 500 D) and middle molecules (MW ≥ 500 D) [1]. Uremia induces the disruption of intestinal epithelial tight junction barriers and alters the composition and metabolic activity of gut microbial flora, resulting in the increased production of noxious toxins that then enter the circulation and cause systemic inflammation [2,3]. In chronic kidney disease (CKD), the decreased diversity of intestinal flora and increased abundance of colonic aerobic bacteria, such as *Enterobacteriaceae* and *Escherichia coli (E. coli)*, result in an unbalanced intestinal ecosystem and the further generation of protein-bound uremic toxins through the proteolysis of undigested proteins retained in the intestine [4]. An analysis of fecal samples from CKD patients showed that phenolic compounds are mainly produced by anaerobic bacteria, whereas indolic compounds are produced by both aerobic and anaerobic bacteria [4]. Indoxyl sulfate (IS), a protein-bound indolic toxin with an MW of 213.21 g mol^−1^, is a metabolite from dietary L-tryptophan amino acid fermentation. In the intestine, *E. coli* tryptophanase enzyme converts tryptophan into indole, which is rapidly absorbed by intestinal epithelial cells and released into the bloodstream. Indole is then hydroxylated to indoxyl and subsequently converted to IS by sulfotransferase enzyme in the liver [5,6,7]. Finally, IS is normally eliminated through the urine via its transport through organic anion transporters (OATs).

The transcellular transport of IS across the cell membrane occurs via the help of OATs. In human renal proximal tubular cells, OAT 1, OAT2 and OAT 3 are located in the basolateral membrane and, driven by the exchange of dicarboxylates, mediate the movement of organic anions from the blood into tubular cells, while OAT4 and OAT10 are located in the apical membrane and facilitate the secretion of organic ions from tubular cells into the urine [8]. OATs are the secondary/tertiary active transporter proteins that are responsible for the elimination of endogenous metabolites (such as IS) and xenobiotics. Wu et al. showed that serum IS levels became higher in OAT1 or OAT3 knockout mice, and these two transporters exerted a synergistic effect in the elimination of solutes or toxins [9]. Additionally, clearance of protein-bound solutes has a near-linear association with the glomerular filtration rate (GFR); therefore, IS clearance is decreased accordingly during GFR decline in CKD progression [10]. According to ultra-performance liquid chromatography–tandem mass spectrometry (UPLC–MS/MS) analysis, serum IS in health participants is ≤0.05–3.02 mg/L, and the average IS level progressively increases from 1.03 in CKD stage 1 to 12.21 mg/L in CKD stage 5 [11]. Moreover, the administration of IS in CKD animals increases IS retention in renal tubular cells and is accompanied by cell death of OAT1- and OAT3-expressing proximal tubular cells, and this effect can be rescued by probenecid, an anion transport inhibitor [12]. Thus, inadequate renal clearance of IS during renal function decline might further aggravate IS-induced renal tubule cytotoxicity and hasten CKD progression. 

AST-120 is an oral charcoal absorbent that functions by adsorbing indole generated in the gastrointestinal tract and thereby lowers serum and urine IS concentrations in CKD. Clinically, adding AST-120 to standard therapy offers a beneficial effect by halting CKD progression, delaying the time to the initiation of dialysis [13]. Among existing clinical data, AST-120 was reported to have a protective role in CKD patients in the post hoc analysis of the Evaluating Prevention of Progression in CKD (EPPIC) trial when combined with RAS blockade in the Carbonaceous Oral Adsorbent’s Effects on Progression of CKD (CAP-KD) study [14]. In addition, some treatments have also been proposed to reduce serum IS accumulation in CKD [15]. Gao et al. reported dietary protein restriction is beneficial in CKD progression, meaning that a low-protein diet (LPD) can greatly lower the serum IS level in CKD and also prevent proteinuria, alleviate renal lesions and retard renal function decline. Supplementation with ketoacids together with LPD improves the weight loss or growth retardation due to LPD [16,17]. Due to the production of IS by gut microbiota, manipulation of gut microbiota is a reasonable proposition to lower the uremic toxin level. Prebiotics, probiotics and synbiotics seem to be the promising options for reducing IS. A randomized trial conducted by Rossi et al. showed that synbiotics could effectively lower p-cresyl sulfate, another uremic toxin, but not IS [18]. In hemodialysis patients, new dialysis techniques have reported the potential benefit in reducing IS levels in ESRD patients by including the use of (1) albumin-binding competitor [19], (2) IS transcellular transporter antagonist [20,21] and (3) hydrophobic and cationic adsorbents with high-flux dialyzers [22].

## 2. Indoxyl Sulfate Induces Tubular Cell Death and Contributes to CKD Progression

**Figure 1:**
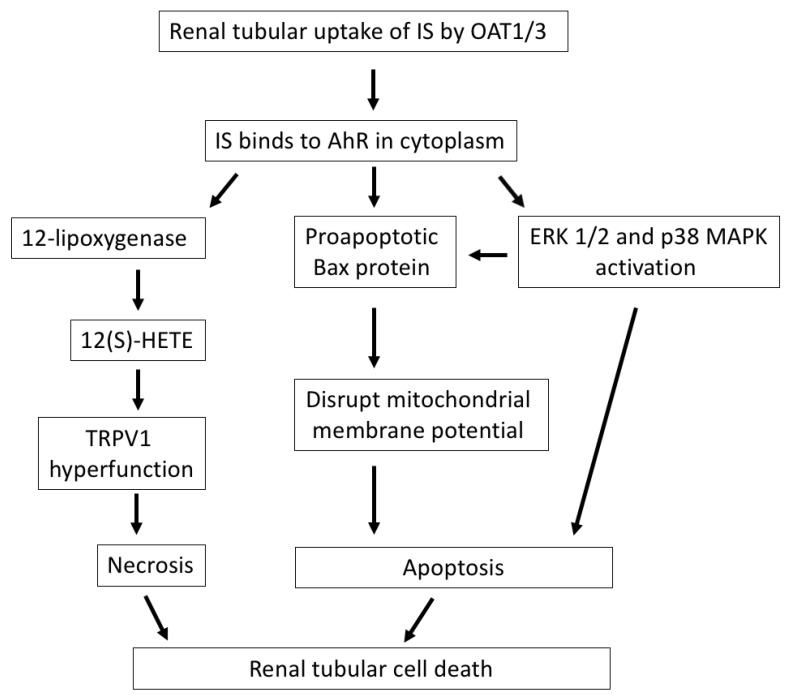
The mechanisms by which IS induces renal tubular cell death. After cellular uptake of IS by OAT1/3 in renal tubules, IS binds to AhR in the cytoplasm and exerts direct tubulotoxicity via necrotic or apoptotic cell death. IS upregulates 12-lipoxygenase and 12(S)-HETE synthesis and induces TRPV1 hyperfunction, which leads to necrosis of tubular cells. Additionally, IS induces apoptosis of tubular cells through the activation of the proapoptotic Bax protein, disrupting the mitochondrial membrane potential, or the activation of ERK 1/2 and p38 MAPK. IS, indoxyl sulfate; OAT, organic anion transporter; AhR, aryl hydrocarbon receptor; 12(S)-HETE, endovanilloid 12-hydroxyeicosatetraenoic acid; TRPV1, transient receptor potential vanilloid 1; Bax, Bcl-2-associated X protein; ERK 1/2, extracellular signal-regulated kinases 1/2; MAPK, mitogen-activated protein kinase.

Renal tubular epithelial cells are easily damaged by a variety of renal insults, such as ischemia and toxin injury, especially in the high-energy-demanding proximal tubular segment. Renal tubular cell death is a direct consequence of acute kidney injury (AKI). In the case of CKD development, either apoptotic or necrotic cell death contributes to tubular injury and renal fibrosis (Figure 1), and the relative contribution of apoptotic and necrotic cell death mechanisms depends on the cause and severity of the renal insults [23]. The extent of tubulointerstitial damage correlates better with renal function decline than with glomerulopathy [24]. Thus, the maladaptive repair of tubular cell injury and interstitial inflammation after kidney damage accelerate CKD progression [25].

Acute tubular necrosis is a major cause of AKI and secondary to acute ischemia or toxin insults [26]. In our previous study, IS was shown to have direct cytotoxicity in renal tubular epithelial cells. After its cellular uptake by OAT, IS induced tubular cell necrosis that was presented by the increase in LDH release and reduction in viability in these cells. The function of transient receptor potential vanilloid 1 (TRPV1) in tubular cells was significantly increased after treating cells with IS via binding to aryl hydrocarbon receptor (AhR). TRPV1 hyperfunction plays a crucial role in mediating IS-induced tubulotoxicity through the upregulation of 12-lipoxygenase and endovanilloid 12-hydroxyeicosatetraenoic acid synthesis [27].

The emerging role of apoptotic cell death of renal tubules is gradually being recognized in ischemia or toxic renal injury. IS treatment triggers typical apoptotic morphological changes in human proximal tubular cells, upregulates proapoptotic Bcl-2-associated X (Bax) protein expression and disrupts mitochondrial metabolic activity [28]. Furthermore, IS induces epithelial-to-mesenchymal transition (EMT) and apoptotic cell death of renal tubular cells through the activation of extracellular signal-regulated kinases 1/2 (ERK 1/2) and p38 mitogen-activated protein kinase (MAPK). The occurrence of EMT and apoptosis in IS-treated renal tubules depends on the degree of IS exposure: a low dose of IS is associated with the inhibition of tubular cell proliferation and related to EMT changes, while a high dose of IS is associated with early apoptosis in in vitro experiments. Both EMT and apoptosis of renal tubular cells impair the capacity of the tubular cells to recover from damage and facilitate the development of CKD [29].

In animals with normal renal function, IS in tubular epithelial cells is only weakly immunohistochemically stained, while in CKD animals, IS is intensively stained in the proximal tubular cells, especially in dilated segments [29]. Besides its role in decreasing serum and urine IS, AST-120 can markedly reduce IS staining in remnant tubular epithelial cells, as well as attenuate tubular injuries, such as tubular dilation and atrophy [30]. In addition, after the administration of AST-120 for 48 weeks in subtotal nephrectomy rats, renal tubular injury was significantly improved with the suppressed gene expression of clusterin, which is closely associated with tubular cell damage [31].

## 3. Indoxyl Sulfate Increases Oxidative Stress and Is Associated with Tubular Injury

Among renal tubules, the proximal renal tubule is a major site of ATP production because of its high ATP and oxygen requirements to support the massive levels of ion transport in epithelial cells. ATP production in renal tubules by glucose oxidative metabolism renders tubular epithelial cells vulnerable to oxidative stress damage. Reactive oxygen species (ROS) production is increased in various inflammatory diseases, such as diabetic nephropathy, uremic toxin-related tubular injury and ischemia–reperfusion injury, and is involved in the initiation and progression of CKD [32,33,34]. The major source of free radical formation in kidneys or vessels is NADPH oxidase (NOX) and mitochondrial oxidative phosphorylation. Low levels of free radicals are beneficial for physiological functions such as cell survival, immune responses to pathogens and various cellular signals [35]. However, the overproduction of free radicals generates oxidative stress and damages cell biomolecules such as lipids, proteins and nucleic acids and consequently causes a broad spectrum of chronic diseases [36]. NOX can react with oxygen and transfer an electron from NADPH to form superoxide, which is then converted to hydrogen peroxide, both of which are free radicals that damage cells. In the human kidney, the distribution of NOX1 to NOX4 is widespread, including the mesangium, macula densa, proximal and distal renal tubules, endothelium and vascular smooth muscle cells [37]. NOX4 is the predominant isoform and is constitutively expressed in the kidney, especially in mitochondria of the kidney cortex [38]. The overproduction of ROS by NOX4 leads to oxidative stress damage in the pathogenesis of diabetic nephropathy and renal function deterioration. ROS derived from the NOX4 catalytic moiety and its subunit p22^phox^ are implicated in the pathogenesis of p-cresyl sulfate (PCS)-induced tubular cells cytotoxicity and also increase the expression of inflammatory cytokines and profibrotic factor TGF-β1 [39]. Furthermore, in 5/6 nephrectomy animals, the accumulation of PCS contributes to renal tubular injury and extracellular matrix (ECM) deposition through NOX4-dependent ROS generation [39]. Thus, NOX4 is a reasonable therapeutic target for inhibition to alleviate or prevent CKD progression.

**Figure 2:**
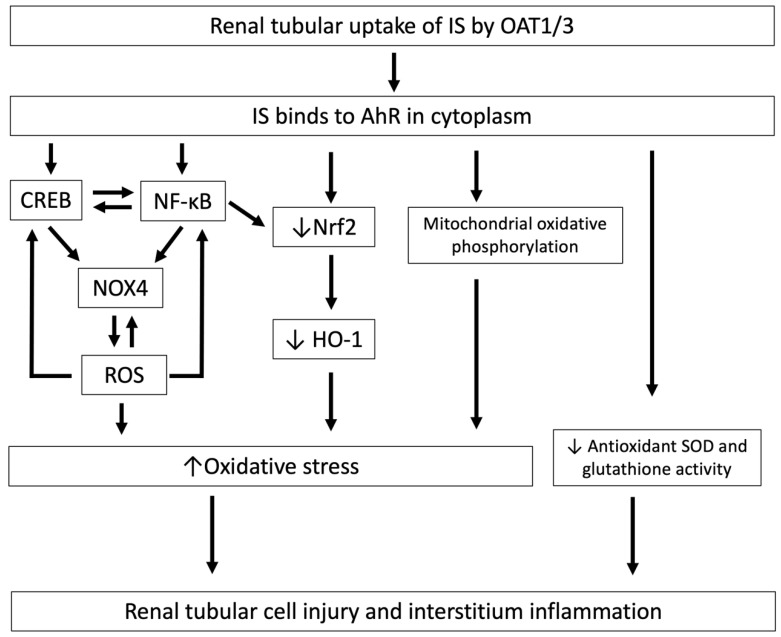
The mechanisms by which IS induces oxidative stress and is associated with tubular cell injury and interstitial inflammation. After cellular uptake of IS by OAT1/3 in renal tubules, IS binds to AhR in the cytoplasm and induces tubular cell injury and interstitial inflammation through the downregulation of Nrf2, and this increased oxidative stress induces CREB and NF-κB expression, resulting in increased NOX4-induced ROS production and mitochondrial oxidative phosphorylation, and decreases antioxidant SOD and glutathione activity. IS, indoxyl sulfate; OAT, organic anion transporter; AhR, aryl hydrocarbon receptor; Nrf2, nuclear factor erythroid 2-related factor 2; NOX4, NADPH oxidase 4; ROS, reactive oxygen species; NF-κB, nuclear factor-κB; CREB, cAMP response element binding protein; SOD, superoxide dismutase.

As with PCS, after the cellular uptake of IS, oxidative stress in HK-2 tubular epithelial cells is greatly increased and is associated with tubulointerstitial injury [40]. As is well known, ROS levels are significantly elevated in renal tubular cells in CKD [37]. ROS promote IS-induced nuclear factor-κB (NF-κB) and cAMP response element-binding protein (CREB) expression in proximal tubular cells. NF-κB and CREB can positively regulate each other, and subsequently promote IS-induced NOX 4 expression. In addition, ROS also promote IS-induced NOX 4 expression in proximal tubular cells. Therefore, ROS, NF-κB and CREB coordinately regulate each other, aggravating the oxidative burden in proximal renal tubules, and thereby play an important role in the pathogenesis of CKD progression [41] (Figure 2). Furthermore, IS downregulates nuclear factor erythroid 2-related factor 2 (Nrf2) through NF-κB activation and the subsequent decrease in antioxidant oxygenase-1 (HO-1) expression, which in turn increases the ROS burden in the cytoplasm of tubular cells [42].

IS also reduces glutathione activity in renal tubular cells; glutathione is known for its powerful antioxidant capacity against oxidative stress [43]. In fact, plasma glutathione levels are reduced in CKD patients and even further decreased if comorbid with diabetes [44,45]. In advanced CKD, serum IS levels are also positively correlated with inflammatory markers that contribute to cardiovascular (CV) and renal toxicity [46]. Notably, superoxide dismutase (SOD) is an enzyme responsible for catalyzing the superoxide radical into molecular oxygen and hydrogen peroxide and functions as a major defender against oxidative stress in renal tubules. In diabetic nephropathy, decreased SOD activity predisposes tubular cells to oxidative stress damage and presents with albuminuria [47]. Among mammalian SOD isoforms, SOD3 is abundant in the kidney and primarily expressed in the extracellular space to protect against superoxide free radical injury, especially in proteinuria kidney disease [48]. Either inadequate SOD1 or SOD3 expression is found to be involved in microvascular and macrovascular complications and is associated with the severity of proteinuria in diabetic nephropathy [49]. Administration of IS to CKD animals for two weeks greatly decreases SOD activity and tubular SOD immunostaining, and the kidney superoxide scavenging activity is markedly reduced as the IS concentration increases [50].

Administration of AST-120 reduces serum IS levels in subtotally nephrectomized CKD rats and is accompanied by the upregulation of Nrf2 and antioxidant HO-1 expression in the cytoplasm of tubular cells, which, in turn, reduces ROS production. Thus, AST-120 has the ability to ameliorate IS-induced oxidative stress in tubular epithelial cells [42]. After 16 weeks of AST-120 treatment in subtotal nephrectomy CKD animals, urinary acrolein, an end-product of lipid peroxidation that is used as a marker of oxidative stress, was significantly decreased [51]. In another study, AST-120 treatment for 34 weeks greatly reduced serum IS concentrations, urinary acrolein and 8-hydroxydeoxyguanosine levels (8-OHdG, a marker of oxidative DNA damage due to exposure to oxygen radicals) and concurrently alleviated myocardial fibrosis and improved left ventricular volume. Thus, AST-120 also enables the amelioration of IS-induced oxidative stress in cardiac cell injury, fibrosis and resultant left ventricular hypertrophy [52]. In CKD patients, daily treatment with 6 g of AST-120 for 12 months significantly decreased urinary 8-OHdG and urinary L-FABP, a marker of tubular damage [53]. Additionally, 24 weeks of AST-120 treatment decreased the increased oxidized glutathione/glutathione ratios in CKD patients, which means that IS can rescue the antioxidant reserve of glutathione [54]. In summary, AST-120 reduces the IS level, alleviating IS-induced oxidative damage on tubular cells.

## 4. Indoxyl Sulfate Stimulates Renal Fibrosis Through TGF-β1 Overproduction

Renal fibrosis is the inevitable consequence of the excessive accumulation of ECM after renal insults, and it is characteristic of glomerular sclerosis, interstitial fibrosis, interstitial infiltration of circulating inflammatory cells such as macrophages, myofibroblast transition of remnant renal cells, fibroblast activation and renal tubular cell apoptosis and atrophy, which ultimately advance CKD progression [55,56,57]. Overproduction of transforming growth factor-β1 (TGF-β1) by injured tubular epithelial cells is a key element in the development of renal fibrosis after renal insults; this is known because tubular atrophy and fibrosis can be ameliorated by targeting TGF-β signaling by using neutralizing antibodies to block TGF-β or anti-TGF-β type II receptors [58,59]. TGF-β1 induces myofibroblast transition from remnant renal cells to facilitate renal fibrosis, although its origin remains controversial, potentially including tubular epithelial cells, endothelial cells, pericytes, fibroblasts or bone marrow-derived cells [60]. After TGF-β1 binds to the TGF-β receptor, TGF-β Receptor I Kinase (TβRI Kinase) phosphorylates and activates selected Smad complexes that translocate into the nucleus to regulate the expression of target genes, such as profibrotic connective tissue growth factor (CTGF), which is a secreted matricellular protein and is associated with fibrotic diseases. In addition, activated TGF-β receptors activate non-Smad pathways to modulate profibrotic responses, such as MAP kinase pathways, phosphatidylinositol-3-kinase/AKT pathways and the Rho-like GTPase signaling pathway, after the activation of different ligands [61]. In normal human kidneys, TGF-β1 expression is mainly located in renal tubular cells but not in glomeruli, while TGF-β2/3 is mainly expressed in podocytes and variably expressed in tubular epithelial cells [62]. TGF-β2 has antifibrotic properties through the upregulation of sphingosine kinase-1 (SK-1) activity, followed by the attenuation of profibrotic CTGF expression and suppression of the fibrotic response [63].

**Figure 3:**
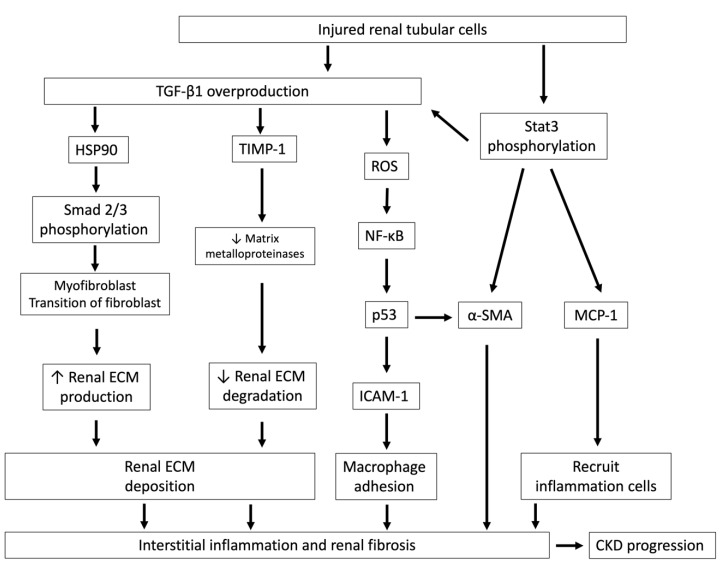
Injured renal tubular cells overproduce TGF-β1 and lead to interstitial inflammation and renal fibrosis. Injured renal tubules increase the overproduction of TGF-β1 and Stat3 phosphorylation and lead to interstitial inflammation and renal fibrosis, which ultimately hasten CKD progression. After TGF-β1 signaling activation, HSP90 and Smad 2/3 are phosphorylated and cause myofibroblast transition of fibroblasts, increase renal ECM production and also reduce TIMP-1 expression, an inhibitor of matrix metalloproteinases, which are responsible for ECM degradation. TGF-β1 also stimulates ROS generation and increases ICAM-1, which is an adhesion molecule that promotes monocyte/macrophage adhesion. The activation of Stat3 phosphorylation in tubules by IS stimulation leads to increases in MCP-1, which recruits inflammatory cells, and tubular α-SMA, which aggravates renal fibrosis. TGF-β1, transforming growth factor-β1; HSP90, heat shock proteins 90; ECM, extracellular matrix; TIMP-1, tissue inhibitor of metalloproteinase-1; ROS, reactive oxygen species; NF-κB, nuclear factor-κB; p53, protein 53; ICAM-1, intercellular adhesion molecule-1; MCP-1, monocyte chemotactic protein-1; α-SMA: α-smooth muscle actin; Stat 3, signal transducers and activators of transcription.

Renal tubular epithelial cells are vulnerable and often the target of various forms of injury. TGF-β signaling in injured renal tubules plays a central role in recruiting inflammatory cells, thereby promoting renal ECM production, myofibroblast phenotypic transition of fibroblasts and further tubular epithelial cell injury [64] (Figure 3). Administration of IS in CKD animals increases the profibrotic factor TGF-β1; tissue inhibitor of metalloproteinase-1 (TIMP-1), an inhibitor to matrix metalloproteinases, which are responsible for ECM degradation; and proα1 (I) collagen, a procollagen precursor expressed in the renal cortex. These changes lead to glomerular sclerosis and interstitial fibrosis [65]. Moreover, IS can increase the production of reactive oxygen species (ROS) and subsequently activates nuclear factor-κB (NF-κB) and p53 in rat proximal renal tubular cells in vitro, thereby promoting the intercellular expression of adhesion molecule-1 (ICAM-1)—an adhesion molecule involved in monocyte/macrophage adhesion—in the cytoplasm of renal tubular cells, a process that is involved in the pathogenesis of interstitial fibrosis [66]. Furthermore, IS promotes the transformation of rat kidney fibroblast cells into the matrix-producing phenotype by upregulating heat shock protein 90 (HSP 90) and the TGF-β/Smad signaling pathway, which then increase collagen I deposition, leading to interstitial fibrosis [67]. Consequently, during renal function decline, the accumulation of IS-damaged renal tubules and damaged renal tubule-secreted TGF-β1 constitute a vicious cycle that underlies the mechanism of IS-induced nephrotoxicity.

Moreover, the Janus kinase family (JAK) and signal transducers and activators of transcription (Stat) pathways have been recently implicated in the pathogenesis of human renal disease. Activation of Stat3 phosphorylation via its increased transcriptional activity in renal tubular cells is recognized as having a central role in the development of renal fibrosis and CKD progression [68]. On the contrary, inhibition of JAK2 and Stat3 in unilateral ureteral obstruction (UUO)-related renal fibrosis could abrogate the myofibroblast phenotypic transition of renal cells and attenuate renal fibrosis to improve renal function [69]. In 5/6 nephrectomy animals, the expression of phosphorylated Stat3 increased as compared with animals with normal renal function. In the same study, an in vitro experiment showed that IS induced the phosphorylation of Stat3 on tyrosine 705 and then increased the expression of inflammatory and fibrotic genes, such as TGF-β1, monocyte chemotactic protein-1 (MCP-1, a potent chemotactic factor for monocytes) and α-smooth muscle actin (α-SMA, an isoform of actin and marker of myofibroblasts) in human proximal tubular HK-2 cells, which are involved in renal inflammation and fibrosis, and may contribute to renal failure [70]. In fact, the degree of interstitial fibrosis is correlated with urinary TGF-β levels in patients with glomerular diseases, and therefore urinary TGF-β is a suspected biomarker that predicts the progression of human glomerular disease [71].

AST-120 reduces the accumulation of IS and retards CKD progression, prolonging the time to the initiation of dialysis [13]. In a dose-ranging study, the use of AST-120 in CKD subjects caused a dose-dependent decrease in IS concentration [72]. In proximal renal tubular cells, IS downregulates the expression of Nrf2—a transcription factor that regulates antioxidant and detoxification genes, such as HO-1—and increases the production of ROS. In a study of unilateral nephrectomy in non-insulin-dependent diabetes mellitus rats, 48 weeks of AST-120 alleviated renal function decline and attenuated glomerular sclerosis, tubular injury and interstitial inflammation and fibrosis. Additionally, AST-120 decreased the immunostaining of TGF-β1, ICAM-1 and MCP-1 in the renal cortex of rats [31,73]. Taken together, AST-120 significantly decreases IS accumulation in CKD animals in a dose-dependent manner and serves a protective role against IS-related oxidative injury and related renal fibrosis.

## 5. Indoxyl Sulfate Activates Mesangial Cell Proliferation and Following Tubular Damage

Mesangial cells function as pericytes and reside close to glomerular endothelial cells. Mesangial cells have characteristics of modified smooth muscle cells to regulate glomerular filtration locally and clear immune complex accumulation during glomerular inflammation [74,75]. In glomerular disease, immune complexes deposited in mesangial cells trigger complement activation and generate several inflammatory mediators to destroy filtration barriers and promote leukocyte adhesion, activation and extravasation. Moreover, some inflammatory mediators alter glomerular capillary wall permeability and result in proteinuria [76]. IS induces mesangial cell proliferation through the induction of cyclooxygenase-2 expression and subsequently increases ECM synthesis and deposition, which can further damage remnant nephrons in CKD [77]. After prolonged exposure to IS, caspase-3 activity is increased and directly induces apoptotic cell death of mesangial cells. As expected, IS also increases the oxidative burden in mesangial cells: IS stimulates extracellular superoxide and intracellular hydroxyl radical production in mesangial cells, which causes glomerular damage in a paracrine fashion [78]. In addition, IS increases intrarenal renin–angiotensin system (RAS) activity in mesangial cells and thereby promotes tubular epithelial changes, such as ECM accumulation, EMT changes and interstitial fibrosis, which are involved in the pathogenesis and progression of CKD [79].

## 6. Clinical Studies of Uremic Toxicity of IS in CKD

Due to the high prevalence of CV disease in CKD patients, higher accumulation of uremic toxins has been suggested to be involved in the pathogenesis and progression of CKD. The level of indolic uremic toxins is correlated with the stage of renal failure with or without dialysis. Indoxyl sulfate and indole-3 acetic acid (IAA) belong to the family of indolic uremic toxins. The IAA level has strong predictive power in detecting mortality and CV events in CKD and is also positively correlated with CRP and malondialdehyde, which are used to evaluate inflammation and oxidative stress [80]. However, IAA fails to predict CV events, mortality or graft survival in transplant patients [81].

In contrast, serum IS levels were higher in patients with CKD progression than those without progression in a cohort study with different CKD stages, and the level was strongly associated with renal progression and all-cause mortality if patients had eGFR < 45 mL/min. Thus, in addition to traditional risk factors, serum IS levels in CKD represent a useful tool to predict renal function deterioration and all-cause mortality, especially in advanced CKD [82]. Additionally, a prospective cohort study conducted by Wang et al. showed that serum IS levels became higher in patients with AKI and were associated with all-cause mortality in patients with AKI requiring hospitalization [83]. Although the relationship between IS levels and clinical outcomes is not clearly verified, the decreased tubular excretion of IS in the setting of tubular injury and decreased expression of OATs is involved in the pathogenesis of IS accumulation during AKI [84].

In end-stage renal disease (ESRD) patients on regular hemodialysis, the removal of IS by dialysis is poor due to its high protein-binding activity. On average, a conventional hemodialysis session reduced serum IS concentrations by only 31.8% [85]. In an analysis from the Japan Dialysis Outcomes and Practice Patterns Study (J-DOPPS), total serum IS levels were significantly associated with all-cause mortality and infection composite events, but the associations with CV events and malignancy events did not reach statistical significance. It is noteworthy that the association between total IS and mortality was stronger in hemodialysis patients with residual kidney function. A possible explanation might be that the residual kidney function determined by low serum IS levels or circulating IS can fully represent tissue IS content if kidney function is not preserved in dialysis patients [86].

Moreover, a prospective study among patients on hemodialysis reported that those with high IS levels experienced a greater incidence of heart failure than those in the low-IS level group, even after adjusting for traditional and uremic-related risk factors [87]. Additionally, serum IS levels also predicted the restenosis and thrombosis rate of arteriovenous fistulas or arteriovenous grafts in hemodialysis patients undergoing angioplasty, with a median follow-up of 32 months [88].

IS levels gradually rise in CKD and even further increase in hemodialysis patients, and they have a positive association with aortic calcification and vascular stiffness [89]. The increase in serum IS levels is linearly correlated with the aortic calcification score by lateral lumbar X-ray and multislice spiral computed tomography. In addition, the relationship between serum IS levels and pulse wave velocity has a positive correlation. Both aortic calcification and vascular stiffness increase the risk of CV events; therefore, the serum IS level is an important contributor to all-cause and CV mortality. After its cellular uptake by OAT3 in the cell membrane, IS can directly stimulate the proliferation of vascular smooth muscle cells (VSMCs) through the activation of mitogen-activated protein kinase [90]. The activation and proliferation of VSMCs are believed to be involved in the development of atherosclerotic lesions and CV complications in CKD patients [90,91]. After kidney transplantation, serum IS drastically decreases within one month after transplant, and this decrease persists for at least one year. However, there was no correlation between IS levels and graft survival, CV events or all-cause mortality [92].

## 7. Conclusions

IS, a protein-bound indolic uremic toxin accumulated in CKD, exerts its tubulotoxicity by directly inducing cell death by either apoptosis or necrosis. IS also increases oxidative stress and decreases antioxidant capacity, which leads to tubular cell injury and interstitium inflammation. The injured renal tubule activates TGF-β1 signaling, drives interstitial inflammation and renal fibrosis in response to induction by IS and is involved in the pathogenesis and progression of CKD. Clinically, the serum IS level is significantly greater in CKD with progression; therefore, its level represents a useful marker to predict renal function decline in CKD patients. Notably, the serum IS level is also independently associated with CV events and all-cause mortality, especially in advanced CKD.

## Abbreviations

8-OHdG	8-hydroxydeoxyguanosine
α-SMA	α-smooth muscle actin
AKI	Acute kidney injury
CV	Cardiovascular
CKD	Chronic kidney disease
EMT	Epithelial-to-mesenchymal transition
ESRD	End-stage renal disease
GFR	Glomerular filtration rate
IAA	Indole-3 acetic acid
ICAM-1	Intercellular expression of adhesion molecule-1
IS	Indoxyl sulfate
JAK	Janus kinase family
LPD	Low-protein diet
MCP-1	Monocyte chemotactic protein-1
MW	Molecular weight
NOX	NADPH oxidase
OAT	Organic anion transporter
ROS	Reactive oxygen species
Stat	Signal transducers and activators of transcription
TIMP-1	Tissue inhibitor of metalloproteinase-1
TRPV1	Transient receptor potential vanilloid 1
VSMCs	Vascular smooth muscle cells
